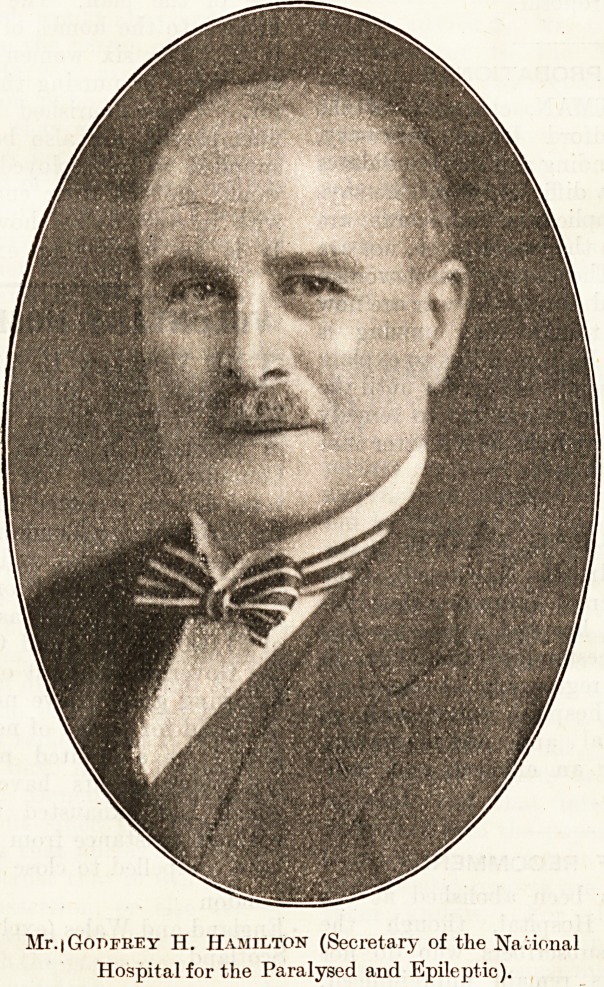# The Hospital and Nursing Exhibition

**Published:** 1923-05

**Authors:** 


### THE HOSPITAL AND NURSING EXHIBITION.

### LECTURES AND CONFERENCES.

THE Hospital, Nursing and Midwifery Exhibition, which was held this year from April 3 to April 7, in the Central Hall, Westminster, was exceptionally successful. The premises were more spacious and the stalls more attractive and more numerous than formerly. At the private view there were many distinguished guests, and throughout the week the Hall was thronged with visitors. The various lectures were much appreciated, and the demonstrations by the British Red Cross were also well attended.

Mr. Godfrey H. Hamilton (Secretary of the National Hospital for the Paralysed and Epileptic).

The conference was opened with a paper by Colonel Nathan Raw on the "Relation of Physical Disease to Insanity." There was, he said, a close relationship between nerves and brain, and the poisons circulating in the blood, caused by disease, might change a patient altogether and render him temporarily insane. Foremost among the diseases causing this lamentable state of affairs were those of the thyroid gland, women being the principal sufferers. A woman suffering in this way might be changed from a sensible person to one nervous, irritable and suspicious, who, unless carefully treated, might become hopelessly insane. In cases of heart disease, when the circulation of the brain becomes interfered with, similar symptoms might develop, and in later stages of tuberculosis the mental condition of the patient was often difficult. Referring to the treatment of puerperal insanity, Colonel Raw said that there should be some place where a patient could be treated without certification, when with proper treatment she could get well in from four to six months. The need had been urged in Parliament, and he hoped that next session would see the establishment of a special hospital on these lines.

At the lecture on "The Effects of the Use of Pituitary Extract in Labour" given by Dr. Aleck Bourne (Obstetric Surgeon to Out-Patients at St. Mary's and Samaritan Hospitals and to In-Patients at Queen Charlotte's Hospital), various lantern slides were shown. These illustrated the results of some original work done by Dr. Bourne and Dr. Burn at Queen Charlotte's Hospital in connection with a Research Committee of the Royal Society of Medicine.

Mr. E. B. Turner, L.R.C.P., F.R.C.S., spoke on "The Medical, Social and Prophylactic Aspects of Venereal Disease." On the social side, he pointed out, this evil results in much loss of time and work through illness when the victims reach the worse stages of the disease. The State is put to great expense, education suffers, and much industrial misery is caused. No marriage should be contracted by a man or woman suffering from venereal disease until he or she can produce a clean bill of health. The young should not be kept in complete ignorance of the facts of life, and the best constructive policy lies in educating the child on right lines. In the course of an interesting lecture on "Nursing in Russia," Miss Dorothy Nicholls, a member of Lady Paget's Mission, said that the hospitals are usually outside a town, and are arranged in blocks in much the same way as the hospitals of the Metropolitan Asylums Board or the London County Council just outside London. The blocks are devoted to the nursing of fevers, tuberculosis, and midwifery, in addition to medical and surgical cases. Internally they are cheerless—all the walls are white, the beds white, and the nurses clad in white. There are no flowers and no tables, and no attempt at any kind of decoration. There is a head sister, but she has not much authority, and exerts but little control in the wards. The sisters are under the control of the medical superintendent, who arranges their appointments to other hospitals, their off duty and their lectures. The blocks are devoted to the nursing of fevers, tuberculosis, and midwifery, in addition to medical and surgical cases. Each patient keeps all his belongings in an open locker by his bed. All bandaging is done in the bandaging room, the patients being taken there on stretchers or wheeled in their beds. This room is kept as sacredly aseptic as the theatre, and while the stretcher patients are away the nurses have an opportunity of turning their mattresses and changing their linen. They are bathed in bathing rooms on specially constructed tables, the work being done by orderlies and sedilkas.

## Figures and Tables

**Figure f1:**